# Supplementary material for: Dynamics of Actin Cytoskeleton and Their Signaling Pathways during Cellular Wound Repair
Source: Cells. 2022 Oct 9;11(19):3166. doi: 10.3390/cells11193166 (PMC9564287; doi:10.3390/cells11193166)
Supplement: Supplementary file 1 [file cells-11-03166-s001.zip › Supplementary Table S1.pdf]

Dynamics of actin cytoskeleton and their signaling pathways during cellular wound repair

Shigehiko Yumura, Md. Shahabe Uddin Talukder, Mst. Shaela Pervin, Md. Istiaq Obaidi Tanvir, Takashi Matsumura, Koushiro Fujimoto, Masahito Tanaka, and Go Itoh

Supplementary Table S1 Descriptions of the examined proteins and their participation for wound repair

Categories	Protein Name	Description	Accumulation of GFP-protein at wound site	Sources of GFP-constructs	Actin accumulation in null cells or in the presence of inhibitor	Sources of mutants or constructs
Actin-related	actin	actin	Yes (GFP-actin, GFP-lifeact)	*1	Aberrant (latrunculin)	
	severin	Ca^{2+}-dependent F-actin severing protein like gelsolin	Yes	*1	Normal (null)	*3
	WASP	Wiscott-Aldrich syndrome protein	Yes	*2	Aberrant (wiscostatin or null)	*2
	Arp3	actin-related protein 3	Yes	*2	Aberrant (CK666 or CK869)	
	MyoA	myosin IA heavy chain, Type I myosin	No	Titus labo	ND	
	MyoB	myosin IB heavy chain, Type I myosin	Yes	Titus labo	ND	
	MyoC	myosin IC heavy chain, Type I myosin	Yes	Titus labo	ND	
	filamin	actin binding protein C, ABP120	Yes	*1	Normal (null)	*2
	ABP34	Actin-bundling protein, ABPB	Yes	*1	ND	
	Cap32	Actin-capping protein 32	Yes	*1	ND	
	fimbrin	calcium-regulated actin bundling protein, p67	Yes	*1	ND	
	α-actinin	Actin-bundling protein, ABPA	Yes	*1	Normal (null)	Noegel labo
	coronin	Regulate actin depolymerization	Yes	*3	Aberrant (null)	*3
	cofilin	Sever actin filaments and bind to actin monomers	Yes	Aizawa labo	ND	
	profilins A and B	G-actin binding proteins	ND		Aberrant (double null)	*3
	WASH	Actin nucleation promoting factor activating Arp2/3	ND		Normal (null)	*2
	EfaA1	elongation factor 1 alpha, ABP50	Disappear	*3	ND	
	Myosin II	Type II myosin heavy chain	Disappear (2)	Uyeda labo, *1	Normal (null)	Manstein labo, *2
	MHCKC	myosin heavy chain kinase C	Disappear (2)	Egelhoff labo	Normal (null)	Egelhoff labo
	SCAR	Suppressor of cAMP receptor mutation, WAVE	No	*2	ND	
	vinculin	vinculin A	No	*3	ND	
	CARMIL	Capping protein, ARP2/3 and Myosin I Linker	Yes	*1	ND	
	GmfA	ADF/cofilin-like domain-containing protein	No	*1	ND	
	Cortexillins A and B	actin bundling protein cortexillin A and B	Disappear (2)	*1	Normal (double null)	*3
	paxillin	paxillin B	No	*3	ND	
	formin A	mDia1-like formin A	No	*3	ND	
	formin family	mDia1-like formin family	ND		Aberrant (SMFH3)	
	DwwA	WW domain-containing protein, interacting with the actin cytoskeleton by binding calmodulin	No	*3	ND	
	CAP	cyclase associated protein, linking with the actin cytoskeleton	No	*3	ND	
Signal-related	GAPA	RasGTPase-activating protein	Disappear (2)	*3	ND	
	PI3 kinases (pikA/pikB/pikC/pikF/pikG)	phosphatidylinositol-4,5-diphosphate 3-kinase 1	ND		Normal (quintuple null)	*2

	PH domain of PI3Kinase	Marker of phosphatidylinositol (3,4,5)-trisphosphate	No	*2	ND	
	PakA	P21-Activated protein Kinase	Disappear (2)		Normal (null)	Iijima labo
	IplA	inositol 1,4,5-trisphosphate receptor-like protein A	ND		Aberrant (null)	*2
	Htt	Huntington's disease protein homolog	ND		Normal (null)	*2
	RacA	Rho GTPase racA	No	*1	ND	
	RacE	Rho GTPase racE	ND		Normal (null)	*2
	Rac family	Rho GTPase rac family	ND		Aberrant (EHT1864)	
	PKB	AKT/PKB protein kinase PkbA	ND		Normal (null)	*2
	AcA	adenylate cyclase A	ND		Normal (null)	*2
	calmodulin	calmodulin	Yes	*1	Aberrant (W7)	
	Phg2	protein serine/threonine kinase Phg2	ND		Normal (null)	*2
	PTEN	Phosphatase and tensin homolog	Disappear (2)	Iijima labo	Aberrant (null)	Iijima labo
	SpkA	Stress-activated Protein Kinase-1	No	*2	ND	
	PLC	phosphoinositide-specific phospholipase C	ND		Aberrant (U73122)	
	PLA2	phospholipase A2	ND		Aberrant (BPB or null)	
	TORC2	TOR complex protein kinase	ND		Aberrant (Torin 1)	
	PKC	protein kinase C, PkcA	ND		Normal (BIM I)	
	ElmoE	engulfment and cell motility ELM family protein E	ND		Aberrant (null)	Jin labo
	GcA and SgcA	membrane bound guanylyl cyclase A / soluble guanylyl cyclase A)	ND		Normal (double null)	*2
	GbpC and GbpD	cGMP-binding protein C / cGMP-binding protein D	ND		Normal (double null)	*2
	TorA	a novel protein with predicted coiled-coil motif near its carboxy terminus (Tortoise)	ND		Aberrant (null)	*2
	RasC and RasG	Ras GTPase RasC / Ras GTPase RasG	ND		Normal (double null)	*2
Membrane-related	DlpA	dynamin-like protein A	Disappear (2)	*1	Aberrant (null)	*2
	DlpB	dynamin-like protein B	ND		Normal (null)	*2
	DymA	dynamin A	No	*1	ND	
	LvsA	large volume sphere mutant A	ND		Aberrant (null)	*2
	clathrin	Clathrin heavy chain	Disappear (2)	O'Halloran labo,*1	Normal (null)	O'Halloran labo,*1
	Tsg101	tumor susceptibility gene 101 protein, ESCRT component	ND		Normal (null)	*1
	PefA	penta EF hand calcium binding protein	Yes (1)	*1	ND	
	Vps4	AAA ATPase domain-containing protein, ESCRT component	Yes (1)	*1	ND	
	annexinC1	Ca- and phospholipid-binding protein	Yes (3)	*1	Aberrant (null)	*1
	annexinC2	Ca- and phospholipid-binding protein	No (3)	*1	ND	*1
	golvesin	Golgi and vesicle associated protein	Disappear (2)	*2	ND	
	calreticulin	Ca-binding protein in the endoplasmic reticulum	No (1)	*2	ND	
	LmpA	lysosomal-associated membrane glycoprotein	No (1)	*2	ND	
	cAR1	cAMP receptor 1 in the cell membrane	No (1)	*3	ND	
Microtubule-related	tubulin	alpha-tubulin	No (1)	*1	Normal (thiabendazole)	
	Kif12	kinesin family member 12	ND		Normal (null)	*2

Yes: accumulated at the wound site, No: Not accumulated, ND: Not determined

*1: Yumura laboratory

*2: DictyBase

*3: NBRP Nenkin

(1): Data from our previous report, Talukder, M.S.U. et al., 2020.

(2): Data from our previous report, Tanvir, M.I.O. et al., 2021.

(3): Data from our previous report, Pervin, M.S. et al., 2018.
