# Supplementary material for: Dynamics of Actin Cytoskeleton and Their Signaling Pathways during Cellular Wound Repair
Source: Cells. 2022 Oct 9;11(19):3166. doi: 10.3390/cells11193166 (PMC9564287; doi:10.3390/cells11193166)
Supplement: Supplementary file 1 [file cells-11-03166-s001.zip › Supplementray Figures.pdf]

Dynamics of actin cytoskeleton and their signaling pathways during cellular wound repair

Shigehiko Yumura, Md. Shahabe Uddin Talukder, Mst. Shaela Pervin, Md. Istiaq Obaidi Tanvir, Takashi Matsumura, Koushiro Fujimoto, Masahito Tanaka, and Go Itoh

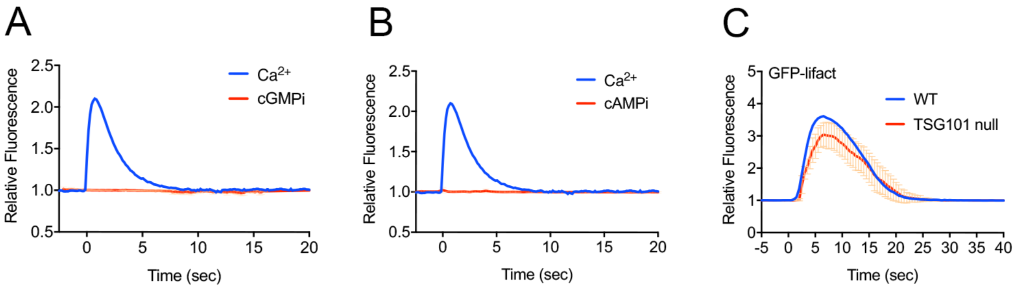

Supplementary Figure S1. Early signals for wound response. (A) Time course of fluorescence intensities in cells expressing Dd-Green cGull, a cGMP sensor, upon wounding (red). Time courses of fluorescence intensities in cells expressing Dd-GCaMP6s, a Ca^{2+} sensor, are also plotted for comparison (blue). (B) Time course of fluorescence intensities in cells expressing Flamindo 2, a cAMP sensor, upon wounding (red). Time courses of fluorescence intensities in cells expressing Dd-GCaMP6s are also plotted for comparison (blue). (C) Time courses of fluorescence intensities at wound sites in TSG101-null (red) or wild-type (blue) cells expressing GFP-lifeact upon wounding ($n \geq 25$ each).

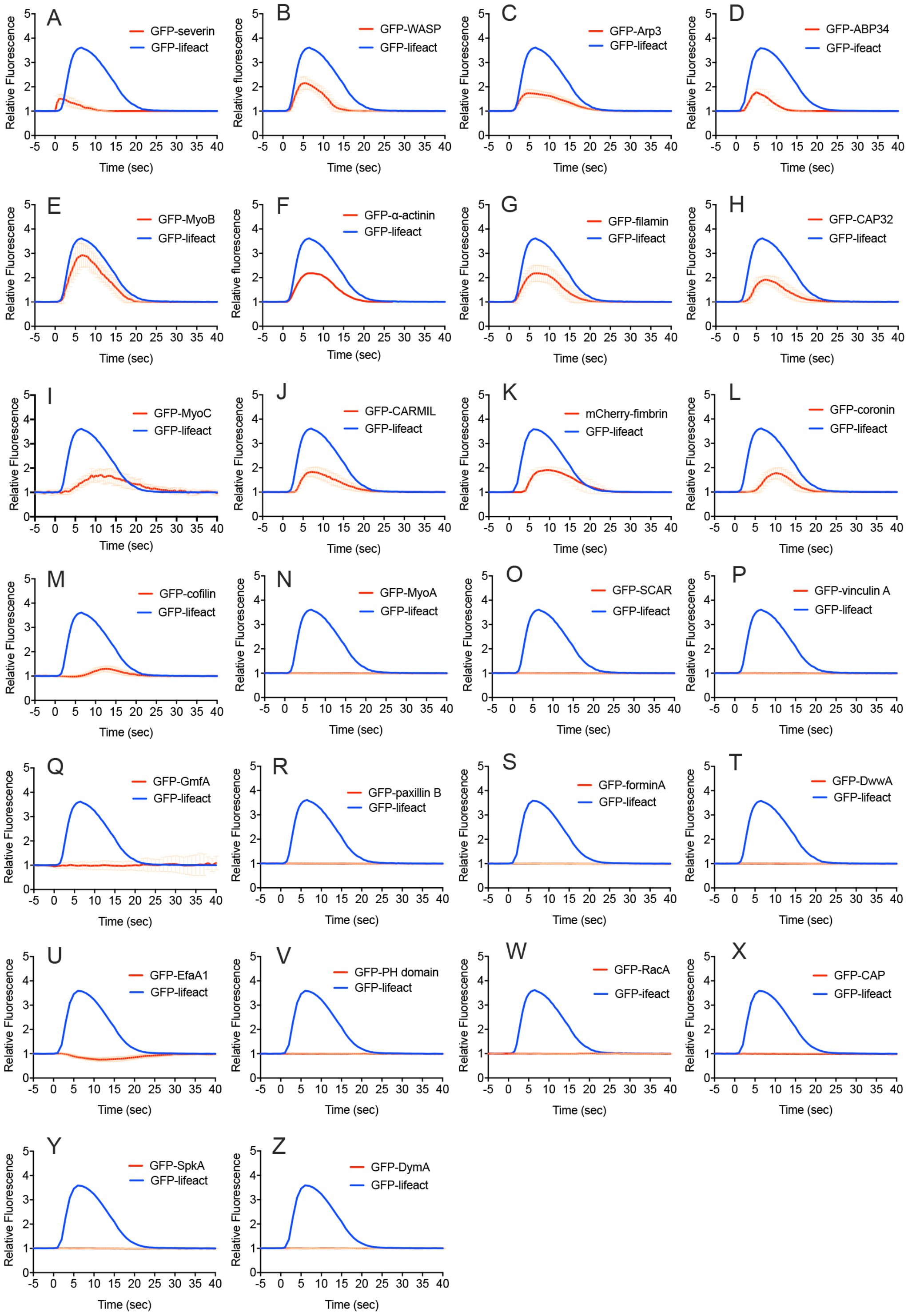

Supplementary Figure S2. Time courses of fluorescence intensities of examined GFP-tagged proteins. Time courses of fluorescence intensities in cells expressing indicated GFP-tagged proteins upon wounding (red). In the same graphs, the time courses of the fluorescence intensities of GFP-lifeact are plotted for comparison (blue). The GFP-tagged proteins (A-M) accumulated at the wound site. (A) GFP-severin, (B) GFP-WASP, (C) GFP-Arp3, (D) GFP-ABP34, (E) GFP-MyoB, (F) GFP-α-actinin, (G) GFP-filamin, (H) GFP-CAP32, (I) GFP-MyoC, (J) GFP-CARMIL, (K) mCherry-fimbrin, (L) GFP-coronin, (M) GFP-cofilin. The other examined GFP-tagged proteins did not accumulate at the wound site. (N) GFP-MyoA, (O) GFP-SCAR, (P) GFP-vinculin A, (Q) GFP-GmfA, (R) GFP-paxillin B, (S) GFP-formin A, (T) GFP-DwwA, (U) GFP-EfaA1, (V) GFP-PH domain, (W) GFP-RacA, (X) GFP-CAP, (Y) GFP-SpkA, and (Z) GFP-DymA ($n \geq 25$ each).

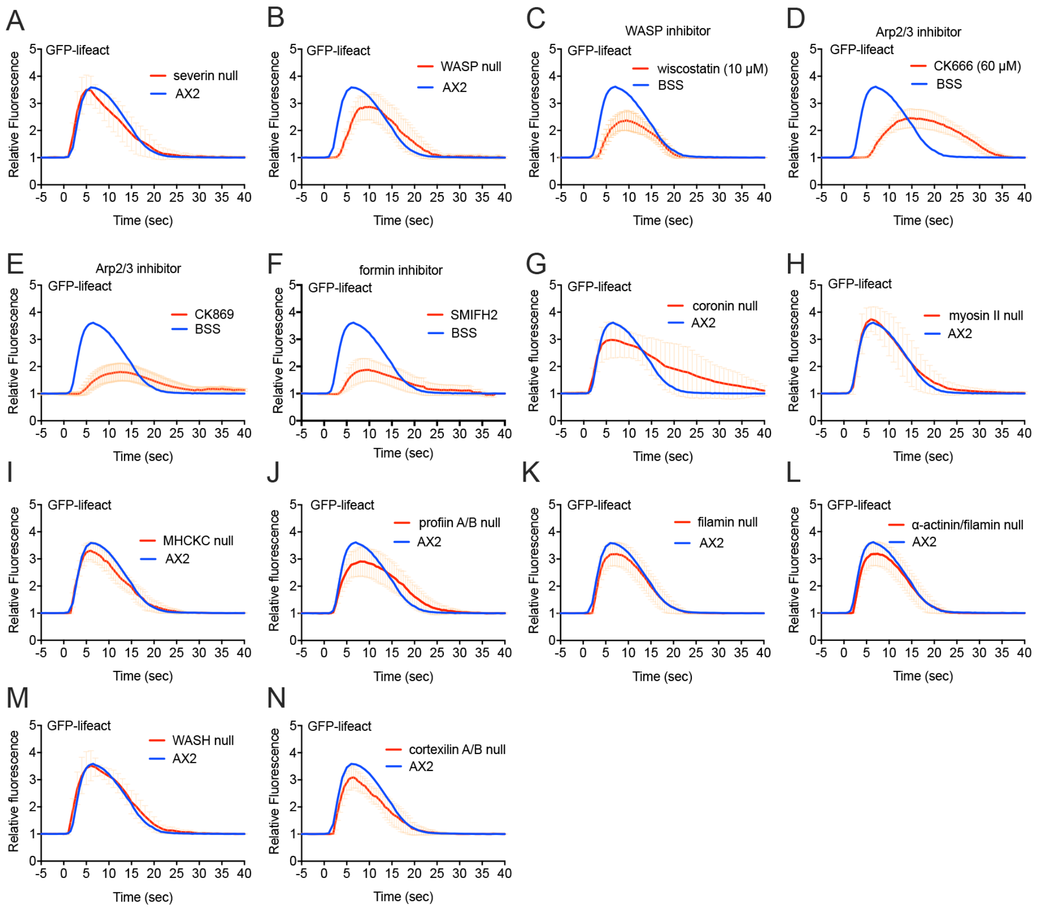

Supplementary Figure S3. Time courses of fluorescence intensities of GFP-lifeact in ARP-null or wild-type cells in the presence of inhibitors. Time courses of fluorescence intensities of GFP-lifeact at wound sites in various ARP-null or wild-type cells in the presence of inhibitors (red). In the same graphs, time courses of the fluorescence intensities of GFP-lifeact in wild-type cells are also plotted as a control (blue). (A) Severin-null cells, (B) WASP-null cells, and (C) wild-type cells in the presence of wiscostatin, (D) wild-type cells in the presence of CK666, (E) wild-type cells in the presence of CK869, (F) wild-type cells in the presence of SMIFH2, (G) coronin-null cells, (H) myosin II-null cells, (I) MHCKC-null cells, (J) profilin A/B double-null cells, (K) filamin-null cells, (L) α-actinin/filamin double-null cells, (M) WASH-null cells, and (N) cortexillin A/B double-null cells ($n \geq 25$ each).

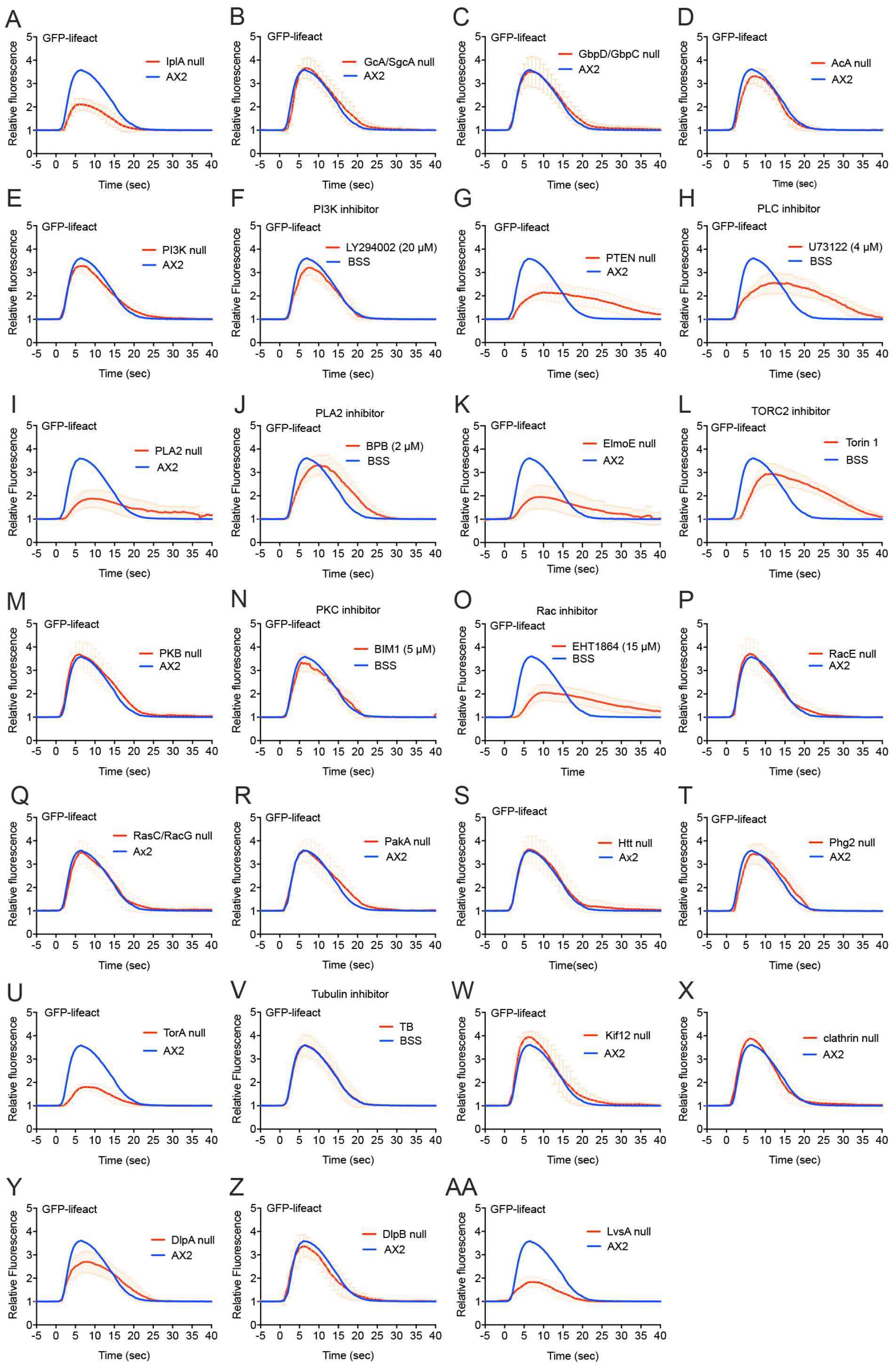

Supplementary Figure S4. Time courses of fluorescence intensities of GFP-lifeact in signal-related or other protein-null or wild-type cells in the presence of inhibitors. Time courses of fluorescence intensities of GFP-lifeact at the wound site in signal-related or other protein-null or wild-type cells in the presence of inhibitors (red). In the same graphs, time courses of the fluorescence intensities of GFP-lifeact in wild-type cells (AX2 or BSS) are also plotted as a control (blue). For the experiments using inhibitors, the inhibitors were dissolved in BSS. (A) IplA-null cells, (B) GcA/SgcA double-null cells, (C) GbpC/GbpD double-null cells, (D) AcA-null cells, (E) PI3K quintuple-null cells, (F) wild-type cells in the presence of LY294002, (G) PTEN-null cells, (H) wild-type cells in the presence of U73122, (I) PLA2-null cells, (J) wild-type cells in the presence of BPB, (K) ElmoE-null cells, (L) wild-type cells in the presence of Torin 1, (M) PKB-null cells, (N) wild-type cells in the presence of BIM1, (O) wild-type cells in the presence of EHT1864, (P) Rac E-null cells, (Q) RasC/RasG double-null cells, (R) PakA-null cells, (S) Htt-null cells, (T) Phg2-null cells, (U) TorA-null cells,

(V) wild-type cells in the presence of TB, (W) Kif12-null cells, (X) clathrin heavy chain-null cells, (Y) DlpA-null cells, (Z) DlpB-null cells, and (AA) LvsA-null cells ($n \geq 25$ each).
